# Effect of Environment on the Evolutionary Trajectories and Growth Characteristics of Antibiotic-Resistant *Escherichia coli* Mutants

**DOI:** 10.3389/fmicb.2019.02001

**Published:** 2019-08-28

**Authors:** Alasdair T. M. Hubbard, Nazila V. Jafari, Nicholas Feasey, Jennifer L. Rohn, Adam P. Roberts

**Affiliations:** ^1^Department of Tropical Disease Biology, Liverpool School of Tropical Medicine, Liverpool, United Kingdom; ^2^Centre for Drugs and Diagnostics, Liverpool School of Tropical Medicine, Liverpool, United Kingdom; ^3^Centre for Urological Biology, Department of Renal Medicine, University College London, London, United Kingdom; ^4^Department of Clinical Sciences, Liverpool School of Tropical Medicine, Liverpool, United Kingdom; ^5^Malawi-Liverpool-Wellcome Trust Clinical Research Programme, University of Malawi, College of Medicine, Blantyre, Malawi

**Keywords:** evolution, antibiotic resistance, fitness, biological cost, urethral organoid, urine

## Abstract

The fitness cost to bacteria of acquisition of resistance determinants is critically under-investigated, and the identification and exploitation of these fitness costs may lead to novel therapeutic strategies that prevent the emergence of antimicrobial resistance. Here we used *Escherichia coli* and amoxicillin–clavulanic acid (AMC) resistance as a model to understand how the artificial environments utilized in studies of bacterial fitness could affect the emergence of resistance and associated fitness costs. Further, we explored the predictive value of this data when strains were grown in the more physiologically relevant environments of urine and urothelial organoids. Resistant *E. coli* isolates were selected for following 24-h exposure to sub-inhibitory concentrations of AMC in either M9, ISO, or LB, followed by growth on LB agar containing AMC. No resistant colonies emerged following growth in M9, whereas resistant isolates were detected from cultures grown in ISO and LB. We observed both within and between media-type variability in the levels of resistance and fitness of the resistant mutants grown in LB. MICs and fitness of these resistant strains in different media (M9, ISO, LB, human urine, and urothelial organoids) showed considerable variation. Media can therefore have a direct effect on the isolation of mutants that confer resistance to AMC and these mutants can exhibit unpredictable MIC and fitness profiles under different growth conditions. This preliminary study highlights the risks in relying on a single culture protocol as a model system to predict the behavior and treatment response of bacteria *in vivo* and highlights the importance of developing comprehensive experimental designs to ensure effective translation of diagnostic procedures to successful clinical outcomes.

## Introduction

The alarming global rise in antimicrobial resistance (AMR) has led to increased interest in resistance development, persistence, and fixation within bacterial populations, with a wide variety of studies published in recent years. These include determining whether the prevalence of resistant bacteria is exacerbated by antimicrobials and their metabolites entering the environment following use in humans (Singer et al., 2016; Caudell et al., 2018), understanding the evolution of AMR due to selective pressure from antimicrobials, and establishing how the acquisition of AMR genes affects bacterial fitness (Hughes and Andersson, 2017; Wong, 2017; Basra et al., 2018). Ultimately, such investigations may inform the design of novel strategies for treating bacterial infection, while preventing the further emergence and spread of AMR in the future.

From an evolutionary perspective, there is a particular focus on the effect of acquisition of AMR on bacterial fitness with a view to suppress the development and persistence of resistance (Palmer et al., 2015; Hughes and Andersson, 2017; Suzuki et al., 2017). When bacteria acquire resistance, either through mutation or horizontal gene transfer, they often incur a fitness cost. Many of these evolutionary studies rely on the *in vitro* development of resistance in the presence of sub-inhibitory concentrations of single, or combinations of, antimicrobials, and investigators employ a range of different media for the generation of resistance (Gullberg et al., 2011; Knöppel et al., 2017; Suzuki et al., 2017; Westhoff et al., 2017) as well as the subsequent assessment of fitness related to acquired AMR (Starikova et al., 2013; Olivares Pacheco et al., 2017). However, the media currently used to grow bacteria *in vitro* is not necessarily a representative model of the *in vivo* environment in which the bacteria live.

Determining evolutionary trajectories to resistance, and the use of this knowledge as a tool to control the emergence and persistence of AMR in bacteria is a growing area of research (Hughes and Andersson, 2017; Wong, 2017; Podnecky et al., 2018). One of the prerequisites for translation of this approach into a clinical intervention that optimizes patient care while leveraging the fitness cost of AMR against a pathogen is the establishment of robust and reproducible model systems which convince clinicians and policy makers that what is seen in the laboratory will actually occur in the real world. Therefore, there is a need to analyze the consequences of different experimental conditions thoroughly and develop model systems which have predictive value in order to inform clinical end-point studies.

A recent study found that mutations that confer antibiotic resistance can arise following evolution in LB broth in the absence of an antimicrobial, raising questions over whether evolutionary trajectories *in vitro* will be the same *in vivo* due to adaption to a specific environment (Knöppel et al., 2017). Therefore, for studies based on *in vitro* model systems to be used to guide changes in treatment and prescription policy or even inform the design of clinical intervention studies, it is critical that experimental observations made in such systems reflect the real-world; as with mathematical models, all models are wrong, but some are useful.

It is known that the fitness cost of a particular mutation conferring resistance to an antibiotic is dependent on the environment it is assessed in, for example growth media (Maharjan and Ferenci, 2017; Lin et al., 2018), and another recent study investigated whether compensatory mutations that arise following acquisition of AMR genes by *Escherichia coli* had the same effect in different media; the results showed that such compensation does not always translate in another environment (Basra et al., 2018). This is an import finding, suggesting that a compensatory mutation can be beneficial in one physiological compartment, such as the bladder, but may be deleterious in another, for example the blood (Basra et al., 2018). It further highlights the importance of the choice of growth media when assessing fitness effects following acquisition of resistance.

*Escherichia coli* is the top urinary pathogen, responsible for upward of 80% of all community-acquired urinary tract infections (UTIs) in otherwise healthy young women (Foxman, 2010). Enhanced sentinel surveillance of *E. coli* bacteremia in England revealed that 51.2% followed UTI with a large number of study isolates found to be resistant to trimethoprim (48.1%), ciprofloxacin (18.5%), and amoxicillin–clavulanic acid (AMC) (45.4%) (Abernethy et al., 2017). Therefore, studying AMR in Enterobacteriaceae with better models is of particular interest as resistance continues to increase within this family to such a degree that carbapenem-resistant, ESBL producing Enterobacteriaceae are now considered priority pathogens on the critical list, for which new drugs are urgently required (World Health Organisation [WHO], 2017).

There have been many *in vitro* models developed to study bacteria that more closely mimic their natural habitats within the human body; these include the chemostat gut, which has been used to study both planktonic and biofilm growth of *Clostridium difficile* (Baines et al., 2005; Crowther et al., 2014), and fermenter models of the human oral cavity, which have been used to study AMR and horizontal gene transfer (Roberts et al., 2001; Ready et al., 2002). More relevant still are laboratory-grown organoids which allow pathogen–host interactions to be investigated and observed in isolation (Forbester et al., 2015; Leslie et al., 2015; Horsley et al., 2018). A recently described urothelial organoid was shown to be tolerant of urine over extended periods of time, to produce a number of expected biomarkers in the presence of urine, and to respond to bacterial infection in a fashion similar to that of the human bladder (Horsley et al., 2018).

In this preliminary study, we sought to determine whether the determination of AMR and the subsequent effect on bacterial fitness is translated to a more physiologically relevant environment. We initially evaluated whether the type of media used during resistance development affects the evolutionary trajectory of the bacterial strain in the presence of sub-inhibitory concentrations of an antibiotic. We chose as a model *E. coli* 10129, a fully susceptible clinical isolate from a patient with clinical suspicion of meningitis in Malawi (Musicha et al., 2017b), a country with high rates of bacteremia caused by *E. coli* (8.8% of bacteremia caused by *E. coli* 1998–2016), many of which are multidrug resistant (Musicha et al., 2017a), including to AMC. For β-lactams to be effective, they need to be maintained at a concentration above the minimum inhibitory concentration for at least 40% of the time between doses in serum (Craig, 1996). However, in 35% of patients AMC was found to be at a concentration above the MIC for <40% of the time (Haeseker et al., 2014). Therefore, the selective pressure during treatment with AMC is often sub-inhibitory, to replicate this we used sub-inhibitory concentrations of AMC to study the development of resistance and subsequent associated fitness costs. We included representatives of different media used in previous evolutionary studies, M9 (defined media) (Suzuki et al., 2017), Iso-Sensitest broth (ISO, semi-defined media) (Farrell et al., 2011), and LB broth (LB, undefined) (Knöppel et al., 2017) and examined whether MICs and fitness determined in growth media could be used to predict the MIC determined in the more physiological environment of urine, and comparative growth in urothelial organoids.

## Materials and Methods

### Bacterial Strains, Media, and Antibiotics

The ancestor bacterial strain used in this study was the clinical isolate *E. coli* 10129 which was isolated from a cerebral spinal fluid (CSF) sample in Malawi (Table 1). It was reported as fully susceptible and the genome had previously been resolved to a relatively small number of contigs within this strain collection (Musicha et al., 2017b).

**TABLE 1:** *Escherichia coli* isolates used during this study.

***Escherichia coli* isolate**	**Comments**	**Origin**
10129	Sensitive clinical Malawian isolate. The ancestral strain used for this study.	Musicha et al., 2017b
ISO_2	AMC-selected derivative of *E. coli* 10129 selected for in Iso-Sensitest broth	This study
ISO_9	AMC-selected derivative of *E. coli* 10129 selected for in Iso-Sensitest broth	This study
ISO_10	AMC-selected derivative of *E. coli* 10129 selected for in Iso-Sensitest broth	This study
LB_1	AMC-selected derivative of *E. coli* 10129 selected for in LB broth	This study
LB_2	AMC-selected derivative of *E. coli* 10129 selected for in LB broth	This study
LB_5	AMC-selected derivative of *E. coli* 10129 selected for in LB broth	This study

Cultures were grown in Mueller Hinton broth (MHB), LB (Lennox) (both Sigma, United Kingdom), ISO (Oxoid, United Kingdom), or M9 [50% (v/v) M9 minimal salts (2×) (Gibco, ThermoFisher Scientific, United States), 0.4% D-glucose, 4 mM magnesium sulfate (both Sigma, United Kingdom), and 0.05 mM calcium chloride (Millipore, United States)]. *E. coli* 10129 and AMC-resistant isolates were grown on LB agar (Lennox) (Sigma, United Kingdom) for colony counting during MIC determination.

Amoxicillin trihydrate:potassium clavulanate (4:1) (AMC) was diluted in molecular grade water (both Sigma, United Kingdom) to a stock concentration of 1 mg/ml and filter sterilized through a 0.22-μM polyethersulfone filter unit (Millipore, United States).

### Determination of Minimum Inhibitory Concentration in Laboratory Growth Media

Minimum inhibitory concentrations were performed in MHB, LB, ISO, or M9, using cultures grown in the respective media in the absence of antimicrobial selection, following the Clinical and Laboratory Standards Institute (CLSI) guidelines for broth microdilution MIC and determined visually (Clinical and Laboratory Standards Institute [CLSI], 2018). As per CLSI guidelines, all cultures were normalized to an optical density at 600 nm (OD_600_) of between 0.8 and 1.0 prior to diluting 1/1000 in the appropriate media. CFU/ml of the inoculum for at each isolate in each media was determined at least once by diluting 1/1000 in phosphate buffered solution (PBS) and 100 μl plated out to ensure that the cell count was between 2 and 8.2 × 10^5^ CFU/ml.

### Determination of Minimum Inhibitory Concentrations in Urine

Overnight bacterial cultures of *E. coli* 10129 and AMC-resistant isolates were diluted in urine supernatants derived from the organoids, with the composition of approximately 25% urine and 75% CnT-Prime 3D Barrier medium (CnT-PR-3D) (CELLnTEC, Switzerland), to OD_600_ of 0.1. Cultures were further diluted 1/1000. Antimicrobial susceptibility was determined by following the CLSI guidelines. Optical density (OD_595_) was measured at 24 h post-incubation with a microplate reader (Biochrom EZ Read 400).

### Selection of Amoxicillin–Clavulanic Acid Resistant *E. coli* 10129

*Escherichia coli* 10129 was grown in sub-inhibitory concentrations (4 μg/ml), as determined by CLSI guidelines in MHB, of AMC in LB, ISO, and M9 to select for resistant isolates. Separate cultures of *E. coli* 10129 were initially grown in 10 ml LB, ISO, or M9 for 18 h at 37°C and shaking at 200 rpm. Following incubation, each culture was serially diluted 1 in 10 in PBS (pH 7.2, Gibco, ThermoFisher Scientific, United States) and 50 μl of the neat to 10^–7^ dilutions were plated out on to LB agar and incubated at 37°C for 18 h to determine CFU/ml. Using the initial cultures, 10 μl was diluted in 10 ml of the respective test media containing 4 μg/ml AMC and incubated at 37°C with shaking at 200 rpm for 24 h. The cultures were serially diluted in PBS as described above and 50 μl of the neat to 10^–7^ dilutions were plated onto LB agar and 50 μl of neat to 10^–2^ dilutions were plated onto LB agar + 8 μg/ml AMC (MIC) and LB agar + 16 μg/ml AMC (2× MIC). These plates were incubated at 37°C for 18 h. Up to 10 single colonies were selected from agar plates from three replicate resistance selection experiments which had growth at the highest concentration of AMC and immediately stored in 1 ml LB + 40% glycerol (Sigma, United Kingdom) and stored at −80°C to prevent any further growth and compensatory mutations. The number of colonies was scored at the dilution that grew between 15 and 100 colonies, except for those that only grew a small number of colonies in the undiluted sample.

### Competitive Fitness Assays

The competitive fitness of each of the selected AMC-resistant isolates was determined in comparison to the ancestral isolate, *E. coli* 10129. Cultures of ancestor and AMC-resistant isolates were initially inoculated in LB, ISO, or M9 directly from the −80°C stocks to minimize any further evolutionary events. The ancestor isolate was grown without the presence of AMC, whereas the resistant isolates were grown in the presence of the same concentration of AMC in which they were initially derived to confirm resistance and ensure that only the resistant population grew, i.e., 8 or 16 μg/ml AMC. All cultures were diluted in the appropriate media to an OD_600_ of 0.1. The AMC-resistant and ancestor isolates were further diluted 1/1000 and combined 1:1 in the same media, 150 μl of which was added to a 96-well plate and incubated at 37°C for 24 h at 200 rpm. The initial combined culture was serially diluted in PBS and 50 μl of the neat to 10^–7^ dilutions was plated out onto both LB agar and LB agar plus either 8 or 16 μg/ml AMC, depending on the concentration of AMC that the isolate was selected on, and incubated at 37°C for 18 h. Following 24 h of growth, the combined culture was then plated out on to agar as described above. The number of colonies was scored at the dilution that grew between 15 and 120 colonies.

Relative fitness was calculated using the Malthusian equation (Lenski et al., 1991):

$$M=M_{\text{m}}\,\left(\frac{T_{24}}{T_{0}}\right)/M_{\text{wt}}\,\left(\frac{T_{24}}{T_{0}}\right)$$

W=(M-1)×100

where *W* is the relative fitness, *M* is the Malthusian parameter, *M*_*wt*_ is the Malthusian parameter of the ancestor isolate, *M*_*m*_ is the Malthusian parameter of the resistant isolate, *T*_0_ is the log CFU/ml count of the initial culture, and *T*_24_ is the log CFU/ml count after 24 h.

### DNA Extraction

Amoxicillin–clavulanic acid-resistant isolates and the ancestral isolate were grown in 10 ml LB and incubated at 37°C with shaking at 200 rpm for 18 h. DNA extraction was performed using the Gentra Puregene Yeast/Bact. Kit (Qiagen, Germany) according to manufacturer’s instructions. The pellet was air-dried at room temperature for 5 min. The DNA was dissolved in 50 μl molecular grade water for 1 h at 65°C and the DNA concentration determined using a NanoDrop^TM^ One/One^*C*^ Microvolume UV–Vis Spectrophotometer (ThermoFisher Scientific, United States).

### Comparative Growth in Urothelial Organoids

Human bladder urothelial organoids were prepared as described previously (Horsley et al., 2018). Briefly, primary human bladder epithelial cell (HBLAK) (CELLnTEC, Switzerland) was seeded onto polycarbonate transwell inserts (VWR, United Kingdom) at 5 × 10^5^ cells per ml in CnT-Prime medium (CnT-PR, CELLnTEC, Switzerland). An appropriate amount of CnT-PR was added to the basolateral chamber of the inserts so that the medium levels were equal, and the cells were submerged. Once cells reached confluency, CnT-PR was replaced with CnT-PR-3D (CELLnTEC, Switzerland) and incubated for 15–16 h. 3D culture was initiated by replacing medium from the apical chamber with filter-sterilized human urine (BIOIVT, United Kingdom), as well as fresh basolateral CnT-PR-3D medium. Urine and CnT-PR-3D medium were changed at regular intervals and organoids were fully established and stratified on days 14–18.

All bacterial cultures of *E. coli* 10129 and AMC-resistant isolates were diluted in urine to OD_600_ of 0.1, then further diluted 1/1000. Urine from apical chambers were aspirated and replaced with diluted bacterial cultures and fresh CnT-PR-3D was added to the basolateral chambers. Inserts were incubated at 37°C, 5% CO_2_ for 24 h. Cultures from apical chambers were collected and transferred into a 96-well plate. Optical density (OD_595_) was measured at time 0 and 24 h post-infection using a microplate reader (Biochrom EZ Read 400).

Background optical density at 595 nm was accounted for by normalizing with a measurement of the urothelial organoids containing the *E. coli* 10129 ancestor or AMC-resistant isolates at 0 h, as well as for organoid containing media only. Relative growth was calculated using the following equation:

$$R=\left(\left(\frac{O_{\text{m}}}{O_{\text{wt}}}\right)-1\right)\,\times \,100$$

where *R* is the relative growth, *O*_*wt*_ is the OD_595_ after 24 h growth of the AMC-resistant isolate in the urothelial organoid, *O*_*m*_ is the OD_595_ after 24 h growth of the ancestor isolate in the urothelial organoid.

### Whole Genome Sequencing and Bioinformatic Analysis

Whole genome sequencing of *E. coli* 10129 and AMC-resistant isolates were performed by MicrobesNG (MicrobesNG, United Kingdom) using 2 × 250 bp paired-end reads on the Illumina MiSeq, which also included the trimming of the sequencing reads.

*De novo* assembly of each of the genomes were performed using SPAdes (version 3.12.0) (Bankevich et al., 2012) and assembly statistics was generated using QUAST (version 4.6.3) (Mikheenko et al., 2018). Annotation of each of the assembled genomes was performed using Prokka (version 1.12) (Seemann, 2014). Single-nucleotide polymorphisms (SNPs), large deletions, mobile genetic element insertions, duplications, amplifications, and smaller insertion and deletion (Indels) sequences in each of the *E. coli* 10129 AMC-resistant isolates were predicated computationally using Breseq (Deatherage and Barrick, 2014) (version 0.32.0) in default mode, with the trimmed sequencing reads produced by MicrobesNG aligned against our assembled and annotated genome of the *E. coli* 10129 ancestor isolate. Any mutations which were predicted with <80% frequency were discounted as were stretches of missing coverage which mapped to small, full contigs from the ancestor. Also discounted were a single 192 bp duplication at the end of a contig from LB_5 and a single amplification of 4 × 96 bp also at the end of a contig from ISO_2 which was deemed an assembly artifact as the sequencing read depth were <20 reads (Supplementary Table S1). We therefore focused only on the SNPs within the genome of the AMC-resistant isolates as predicted by Breseq.

The ancestral isolate, *E. coli* 10129, was characterized bioinformatically to determine the sequence type [MLST 2.0, version 2.0.1 (Larsen et al., 2012)], serogroup [SerotypeFinder, version 2.0.1 (Joensen et al., 2015)], resistome [ResFinder, version 3.1.0 (Zankari et al., 2012)], and plasmidome [PlasmidFinder, version 2.0.1 (Carattoli et al., 2014)] all on default settings.

### PCR Confirmation of Predicted Single-Nucleotide Polymorphisms

Primers were designed to amplify a 465bp region of *cpxA* containing the predicted SNP and a 442-bp region that contained the predicted SNP within the *ampC* promoter region (Table 2). The PCR amplification was performed using the Q5^®^ High-Fidelity polymerase (New England Biolabs, United States) and the final reaction contained 1× Q5^®^ reaction buffer, 200 μM dNTPs, 0.5 μM of the appropriate forward and reverse primers listed in Table 2, and 0.02 U/μl Q5^®^ polymerase in a total volume of 25 μl. The PCR samples were run using the following protocol: denaturation at 98°C for 30 s, followed by 35 cycles of denaturation at 98°C for 10 s, annealing at 69 (*cpxA* SNP) or 67°C (*ampC* SNP) for 30 s, elongation at 72°C for 20 s, followed by a final extension of 2 min at 72°C. The PCR products were cleaned up using the Monarch^®^ PCR and DNA clean-up kit (New England Biolabs, United States). The PCR samples were mixed with 125 μl of the DNA Clean-Up Binding Buffer, transferred to the Clean-Up Columns and centrifuged at 11,400 × *g* for 1 min. The bound DNA was washed with 200 μl DNA Wash Buffer and centrifuged at 11,400 × *g* for 1 min, twice, followed by elution in 20 μl molecular grade water (Sigma, United Kingdom) by centrifuging at 11,400 × *g* for 1 min after incubating at room temperature for 2 min. All PCR products were Sanger sequenced at GeneWiz (Takely, United Kingdom).

**TABLE 2:** Primer names and primer sequences for confirmation of the SNPs in *cpxA* and *ampC* promoter region of *E. coli* 10129 ancestor and AMC-resistant isolates as predicted by breseq.

**Target**	**Primer name**	**Primer sequence**
*cpxA* SNP	Ec10129_F1	TGAACGCAGCGAAATGCAGA
	Ec10129_R1	GTGCGCAGTTCGTGAGAGAT
*ampC* promoter SNP	Ec10129_F2	GGTATTCTGCTGCCGCTAGG
	Ec10129_R2	CCGGGGATCTTTTGTTGCTC

### Biofilm Assay

*Escherichia coli* 10129 ancestor and AMC-resistant isolates were grown in LB for 18 h at 37°C and 200 rpm and then diluted 1/1000 in M9. Three 100 μl technical replicates of the each of the diluted cultures were added to a 96-well microtiter plate alongside three technical replicates of M9 only and three empty wells and incubated statically at 37°C for 24 h. Following incubation, all culture or media was removed, and the wells washed four to five times with 150 μl PBS and then left to dry upside-down for 10 min. The wells were then stained with 125 μl 0.1% Gram’s crystal violet solution (Sigma, United Kingdom) for 15 min at room temperature, then washed four to five times with 150 μl PBS and left to dry upside-down for between 60 and 90 min. The remaining stain was dissolved with 125 μl 30% acetic acid (Sigma, United Kingdom) and incubated at room temperature for 15 min. Finally, the acetic acid solution was transferred to a fresh 96-well microtiter plate and measured at an optical density of 550 nm, with 30% acetic acid used as a blank and the stained empty wells as background.

### Statistical Analysis

Statistical analysis of the initial growth of *E. coli* 10129 in LB, ISO, and M9 was performed using ordinary one-way ANOVA in GraphPad Prism (version 8.0.0). Statistical analysis of competitive fitness in LB, relative fitness in bladder organoids and biofilm production of the *E. coli* 10129 ancestor, and AMC-resistant isolates was performed using ordinary one-way ANOVA plus uncorrected Fisher’s LSD test in GraphPad Prism. Statistical analysis of competitive fitness of LB_2 and LB_5 in LB, ISO, and M9 was performed using unpaired *t*-test. Finally, statistical analysis of cell density of *E. coli* 10129 following challenge with AMC in LB, ISO, and M9 was performed using two-way ANOVA plus uncorrected Fisher’s LSD test, also in GraphPad Prism.

## Results

### Characterization of *E. coli* 10129

*Escherichia coli* 10129 is a clinical isolate from Malawi and is part of the phylogroup B2 (Musicha et al., 2017b), sequence type 700, and serotype O83:H7. The isolate was found to contain the following acquired resistance genes; *sul2* (sulfonamide), *dfrA1* (trimethoprim), *aadA1*, *aph(3′)-Ia*, *aph(6)-Id*, and *aph(3^″^)-Ib* (all aminoglycosides), and *mdf(A)* (macrolide, lincosamide, and streptogramin B). Replicon sequences of IncFII (pRSB107), IncFIA, and IncFIB were detected within the genome sequence, as well as 66.5% coverage of the replicon sequence of IncQ1.

### Selection of *E. coli* 10129 Resistant Isolates

The MIC of AMC for *E. coli* 10129 was determined to be 4–8 μg/ml (Table 3). To select for isolates showing decreased susceptibility to AMC, *E. coli* 10129 was grown in the presence of sub-inhibitory concentrations (4 μg/ml) of AMC in defined (M9), semi-defined (ISO), and undefined (LB) media for 24 h.

**TABLE 3:** Minimum inhibitory concentrations of the *E. coli* 10129 ancestor and AMC-resistant isolates assessed in MHB, LB, ISO, M9, and urine following the CLSI guidelines.

**Medium**	***E. coli* 10129 (μg/ml)**	**ISO_2 (μg/ml)**	**ISO_9 (μg/ml)**	**ISO_10 (μg/ml)**	**LB_1 (μg/ml)**	**LB_2 (μg/ml)**	**LB_5 (μg/ml)**
MHB	4–8	16	8–16	8–16	32	64	64
LB	4–8	16	–	–	–	64–128	64
ISO	8	16–32	–	–	–	128	64–128
M9	1–2	2–4	–	–	–	32	16
Urine	2	4	–	–	–	64	64

**FIGURE 1:**
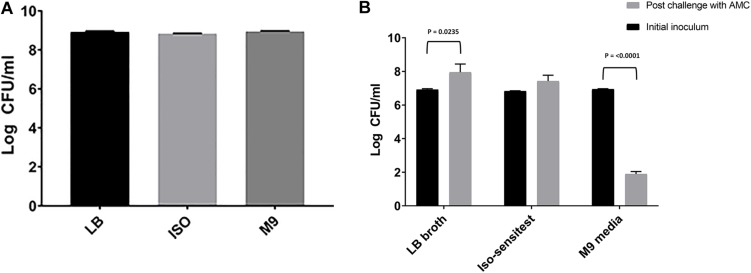
Difference in log CFU/ml of *E. coli* 10129 **(A)** after growth in LB, ISO, and M9, and **(B)** following 24-h exposure with sub-inhibitory concentrations of AMC in LB, ISO, and M9 compared to the initial inoculum. Error bars represent standard error of the mean.

There was no significant difference in the cell densities following growth of *E. coli* 10129 in the absence of AMC among three different media types (*P*-value = 0.1071, Figure 1A) and therefore the cell density of the inoculum was also comparable. A significant reduction in cell density of 5.049 log CFU/ml was observed following incubation in M9 containing AMC (*P*-value < 0.0001, Figure 1B) and we did not recover any isolates from agar containing 8 or 16 μg/ml AMC. Unlike M9, following exposure of *E. coli* 10129 to AMC in ISO there was a small but insignificant increase in cell density of 0.592 log CFU/ml (*P*-value = 0.1339, Figure 1B) and significant growth of *E. coli* 10129 of 1.03 log CFU/ml in LB in the presence of AMC compared with the initial inoculum (*P*-value = 0.0235). Therefore, using this one-step selection technique, 10 colonies were selected from agar plates containing 8 μg/ml AMC and 10 from plates containing 16 μg/ml AMC following growth in ISO and LB broth, respectively.

Of the 10 resistant isolates of *E. coli* 10129 grown in either LB or ISO, 3 from each media-type were selected at random. The three isolates selected following growth in ISO broth were designated ISO_2, ISO_9, and ISO_10 and those grown in LB were designated LB_1, LB_2, and LB_5. ISO_2 and LB_5 were both derived from independent lineages, whereas both LB_1 and LB_2 were possibly derived from the same lineage and ISO_9 and ISO_10 were also possibly from the same lineage.

### Minimum Inhibitory Concentrations of *E. coli* 10129 Ancestor and AMC-Resistant Isolates in MHB

Resistance to AMC in the six selected *E. coli* 10129 derivatives was assessed through MIC determination in comparison to the ancestral isolate in MHB (Table 3). While there was within media-type increased variability in the AMC MIC of the resistant isolates, there was a clear distinction between those resistant isolates selected for in LB and those from ISO (Table 3). There was only a one to threefold increase in the AMC MIC in resistant isolates selected in ISO, which would be expected as ISO selected isolates where only recovered from agar containing MIC concentrations of AMC. The AMC-resistant isolates selected for in LB broth plated out on LB agar containing 2× MIC concentrations of AMC were all highly resistant compared with the ancestor isolate, resulting in a 3- to 15-fold increase in MIC (Table 3). The most resistant isolates were determined to be LB_2 and LB_5, both with a 7- to 15-fold increase in MIC in comparison to the ancestral isolate (Table 3). Four of the six selected isolates had MICs above the clinical breakpoints according to the EUCAST guidelines for systemic infections caused by *E. coli* (>8 μg/ml, Table 3) and therefore deemed AMC-resistant, these were ISO_2, LB_1, LB_2, and LB_5, and were carried forward for whole genome sequencing.

### Single-Nucleotide Polymorphism Prediction

Single-nucleotide polymorphisms that arose in the genomes of the *E. coli* 10129 AMC-resistant isolates following selection in sub-inhibitory concentrations of AMC were identified using Breseq, which predicts SNPs in genomes of derivative generations by mapping sequencing reads to the original ancestral isolate (Deatherage and Barrick, 2014).

Chromosomal mutations of *E. coli* that lead to resistance to β-lactams are often found within the promoter region of *ampC* resulting in over-production of the chromosomally located β-lactamase AmpC (Siu et al., 2003; Tracz et al., 2005, 2007). Isolates LB_2 and LB_5 contained a predicted SNP at nucleotide position-32 in the -35-box promoter region with 100% frequency within the sequencing reads, which was subsequently confirmed to be present using PCR and sequencing. This mutation converted the natural weak -35 promoter (TTGTCA) to a stronger promoter (TTGACA) (Caroff et al., 2000) which has been previously linked to 21-fold increase in AmpC production (Jaurin et al., 1982).

A second SNP with a 100% frequency was predicted in isolate ISO_2, although there was only a modest increase in AMC MIC when assessed in MHB. This SNP occurred in *cpxA*, which encodes a change in amino acid from a proline to leucine at position 177. CpxA is the sensor histidine kinase portion of the envelope stress response two-component system, CpxAR. The CpxAR two-component system is involved in the regulation of several proteins including the efflux pumps *acrB*, *acrD*, and *eefB* (Srinivasan et al., 2012) and the outer membrane porins *ompF* and *ompC* (Batchelor et al., 2005) in a wide range of Enterobacteriaceae including *E. coli*. Therefore, the SNP predicted by Breseq, and subsequently confirmed via PCR and sequencing, present in *cpxA* may explain the modest increase in AMC resistance in ISO_2.

In LB_5, two synonymous SNPs were predicted with 80–90% frequency in *vgrG1*, which is part of the type VI secretion system of Gram-negative bacteria and a significant component for the delivery of effector molecules to other cells (Cianfanelli et al., 2016). One SNP was found in *vgrG1* which mapped to amino acid position 368 (proline) and a second at amino acid 387 (glycine). The SNP in *vgrG1*, and indeed the only predicted SNP, corresponding to amino acid position 368 was also present in AMC-resistant isolate in LB_1 (Supplementary Table S1) with a frequency of 89.1%.

### Minimum Inhibitory Concentrations of *E. coli* 10129 Ancestor and AMC-Resistant Isolates in Other Media and Urine

**FIGURE 2:**
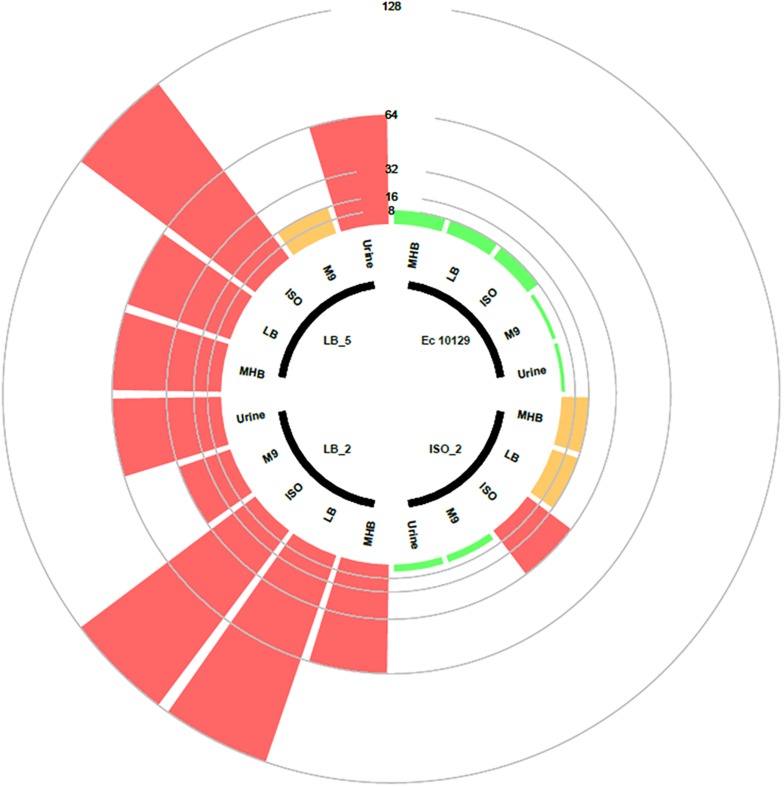
Circular barplot chart displaying the MIC values of the *E. coli* 10129 ancestor with three evolved derivative strains. The inner circle shows which bars belong to which strains and which media the MICs were tested. Clinical breakpoint for sensitivity and resistance is 8 and 16 μg/ml, respectively, and is indicated by a circle. The scale represents the MIC value.

Following observed differences between media types in the selection of AMC-resistant isolates of *E. coli* 10129, we assessed the MIC of the ancestor and AMC-resistant isolates in LB, ISO, and M9 to determine whether media affects AMR (Figure 2). MICs of both the ancestor and AMC-resistant isolates were comparable when assessed in MHB and LB; however, there was a slight increase in MIC in the ancestor isolate and ISO_2, LB_2, and LB_5 when assessed in ISO (Table 3 and Figure 2). There was a considerable decrease in MIC of all isolates when assessed in M9 compared with MHB, LB, and ISO (Table 3 and Figure 2), which means the MIC of AMC in M9 (1–2 μg/ml) is actually lower than the sub-inhibitory concentrations of AMC used to select AMR-resistant isolates in M9 and therefore causing cell death instead of selecting spontaneous AMC-resistant mutants (Table 3 and Figure 2). Therefore, the concentration of AMC used to select AMC-resistant isolates in M9 was not sub-inhibitory but rather bactericidal, explaining the observed decrease in cell density after exposure to AMC in M9 (Figure 1B).

In order to make *in vitro* model systems clinically relevant, more sophisticated model growth environments than an agar plate or tube of media are required. We therefore wanted to ascertain if the observed variability in the growth of the AMC-resistant *E. coli* isolates described above is replicated in urine and with organoids. We found that although the growth of *E. coli* 10129 was the same or better than that of the highly virulent uropathogenic *E. coli* strain UTI89 (Anderson et al., 2003) in 100% urine (data not shown), pure urine is nutrient-poor and overall growth was not robust enough to determine MIC. We therefore used supernatant derived from the apical chamber of urothelial organoids, which consist of urine enriched with cellular exudate and which is more likely to be representative of the host/pathogen interface in the bladder (Horsley et al., 2018). MICs of the ancestor isolate, and the AMC-resistant isolate selected in ISO, assessed in this urine were noticeably lower than those assessed in MHB, LB, and ISO, and were more comparable to those assessed in M9 (Table 3 and Figure 2). The MIC of the AMC-resistant isolates selected for in LB assessed in urine was markedly higher than those assessed in M9 and are in fact more comparable to the MICs assessed in MHB. Taken together, these data suggest that culture environment can have an unpredictable effect on MIC.

### Competitive Fitness of AMC-Resistant Isolates in LB, ISO, and M9

**FIGURE 3:**
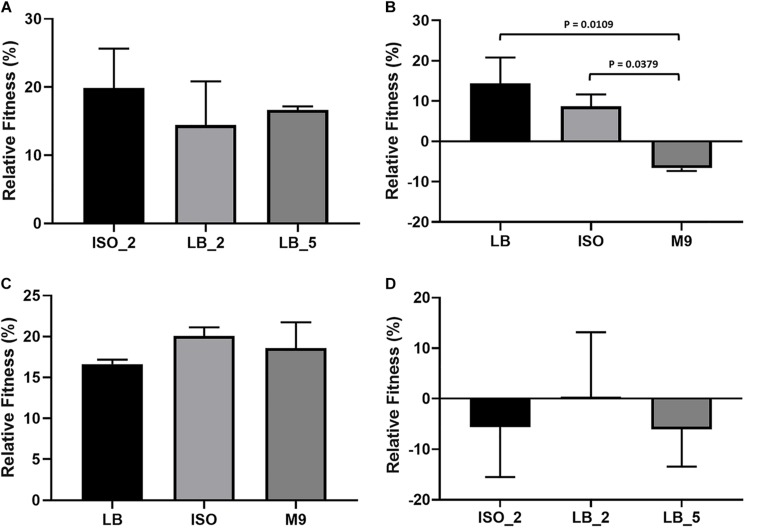
Relative fitness and growth of *E. coli* isolates; error bars represent standard error of the mean. **(A)** Relative fitness of *E. coli* 10129 AMC-resistant isolates ISO_2, LB_2, and LB_5 compared to the ancestral isolate in LB. **(B)** Relative fitness of *E. coli* 10129 AMC-resistant isolate LB_2 in LB, ISO, and M9 compared to the ancestral isolate in the absence of AMC. **(C)** Relative fitness of *E. coli* 10129 AMC-resistant isolate LB_5 in LB, ISO, and M9 compared to the ancestral isolate in the absence of AMC. **(D)** Relative fitness of *E. coli* 10129 AMC-resistant isolates ISO_2, LB_2, and LB_5 compared to the ancestral isolate in urothelial organoids.

The relative fitness of three confirmed *E. coli* 10129 AMC-resistant isolates (ISO_2, LB_2, and LB_5), with identifiable SNPs with 100% frequency and all from independent lineages were initially assessed competitively in LB with the ancestral isolate. There was a significant increase in the fitness of all three AMC-resistant isolates when assessed in LB. ISO_2 increased by 19.9% (*P*-value = 0.0118; Figure 3A), LB_2 increased by 14.4% (*P*-value = 0.0463; Figure 3A), and finally LB_5 increased by 16.6% (*P*-value = 0.0263; Figure 3A) relative to the *E. coli* 10129.

As we have been able to show that media has an important effect on both the selection of *E. coli* 10129 AMC-resistant isolates and the MIC of the ancestor and resistant isolates, we determined the relative fitness of the highly resistant and fit LB_2 and LB_5 isolates which contained the same SNP in the AmpC promoter region in three media types in the absence of AMC: LB, ISO, and M9. There was no significant difference in relative fitness when LB_5 was assessed in LB, ISO, and M9 (*P*-value = 0.4943; Figure 3C) and no significant difference between the relative fitness of LB_2 and LB_5 when compared in LB (*P*-value = 0.7470; Figure 3A). However, the relative fitness of LB_2 was significantly different when assessed in M9 media compared with its growth in ISO (*P*-value = 0.0379) and LB (*P*-value = 0.0109) (Figure 3B). Additionally, there was a significant difference in relative fitness between LB_2 and LB_5 when assessed in either ISO (*P*-value = 0.0216) or M9 (*P*-value = 0.0015) (Figures 3B,C). These data suggest that media choice can also affect fitness.

### Comparative Growth Rates in Urothelial Organoids

As many studies into the evolution or acquisition of AMR and subsequent effects on fitness are ultimately intended to be translated into clinical interventions, we determined whether relative fitness in LB can be used to predict relative growth of the *E. coli* 10129 AMC-resistant isolates compared with the ancestral isolate in urothelial organoids. Despite relative growth varying among the AMC-resistant isolates, no significant difference in relative growth among all three AMC-resistant isolates was observed (*P*-value = 0.8892) (Figure 3D) and, in direct contrast to relative fitness assessed in LB, two out of three AMC-resistant isolates grew slower relative to the ancestral isolate. The relative growth of ISO_2 was 5.6% slower compared to the ancestral isolate despite previously being found to have acquired the largest increase in fitness (19.9%) of the AMC-resistant isolates in LB (Figures 3A,D). Although LB_2 and LB_5 increased in fitness in LB (14.4 and 16.6%, respectively), and in the case of LB_5 an increase in fitness in ISO and M9 as well, LB_2 grew 0.3% faster and LB_5 grew 6.1% slower relative to the ancestral isolate in the urothelial organoid (Table 4 and Figures 3A–D). This indicates that relative fitness assessed in growth media, and in particular LB, has no predictive value to the relative growth in more biologically relevant environments.

**TABLE 4:** Summary table of relative fitness assessed of the *E. coli* 10129 AMC-resistant derivatives compared to the ancestral isolate in LB, ISO, M9, and urothelial organoids; biofilm production measured as optical density at 550 nm; and the nucleotide, amino acid position, and gene in which there were identified SNPs.

	**ISO_2**	**LB_2**	**LB_5**
LB	19.9 ± 5.8	14.4 ± 6.4	16.6 ± 0.5
ISO	–	8.7 ± 2.9	20.1 ± 1
M9	–	−6.6 ± 0.7	18.6 ± 3.2
Urothelial organoids	−5.6 ± 9.9	0.3 ± 12.8	−6.1 ± 7.4
Biofilm production	0.101 ± 0.008	0.118 ± 0.01	0.054 ± 0.002
SNP	CCG → CTG	GTC → GAC	GTC → GAC
Amino acid position	P177L	V111D	V111D
Gene	*cpxA*	*ampC* protomoter	*ampC* protomoter

*Error represents the standard error of the mean.*

### Biofilm Production

**FIGURE 4:**
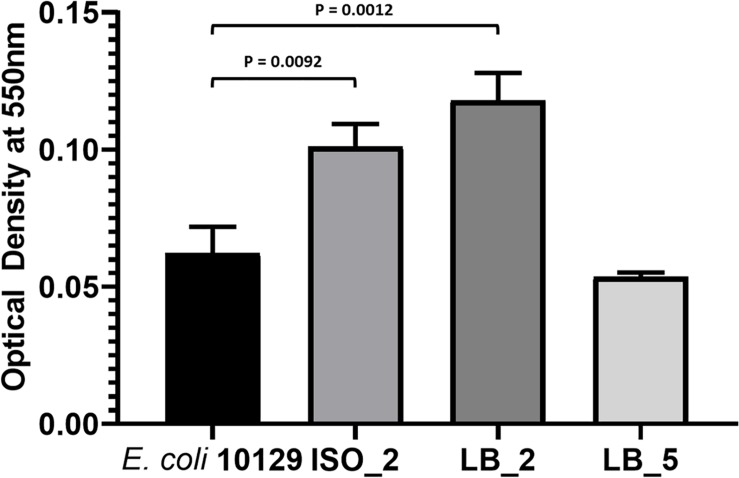
Biofilm production of *E. coli* 10129 ancestor and AMC-resistant isolates in M9. Error bars represent standard error of the mean.

Urinary tract infection isolates often form extensive biofilms on catheter surfaces and we wanted to determine whether the fitness of *E. coli* 10129 AMC-resistant isolates was accompanied by an increase in biofilm production. While biofilm production varied in the AMC-resistant isolates, there was a significant increase in biofilm production for all AMC-resistant isolates in comparison to the ancestor isolate except for LB_5 (*P*-value = 0.4677), which was previously found to acquire a fitness benefit. There was a significant increase in biofilm production compared with the ancestor isolate in both ISO_2 (1.63-fold increase, *P*-value = 0.0092) and LB_2 (1.90-fold increase, *P*-value = 0.0012) (Table 4 and Figure 4), even though ISO_2 grew more slowly (5.6%) and LB_2 marginally grew faster (0.3%) than the ancestral isolate in urothelial organoids. Therefore, we found no correlation between the fitness of AMC-resistant isolates in different growth conditions and biofilm production.

## Discussion

In this preliminary study, we generated resistant isolates in the laboratory using a fully susceptible Malawian clinical isolate *E. coli* 10129 (Musicha et al., 2017b) by exposing it to AMC *in vitro* as this is a geographically and clinically relevant first line treatment for Enterobacteriaceae infections (Musicha et al., 2017a). We used a single-step procedure in three different media types that are commonly used in evolutionary studies (Farrell et al., 2011; Knöppel et al., 2017; Suzuki et al., 2017) to select for AMC-resistant *E. coli* 10129 isolates with AMC-resistance which were then tested for relative fitness compared with the ancestral strain (Lenski et al., 1991; Farrell et al., 2011; Knöppel et al., 2017; Basra et al., 2018). We were able to determine multiple mutations leading to AMC resistance such as a well-characterized SNPs in the *ampC* promoter region (Jaurin et al., 1982; Caroff et al., 2000; Siu et al., 2003; Tracz et al., 2005, 2007), and a less well-defined evidence of AMC resistance including a SNP in *cpxA* (Suzuki et al., 2014; Dean et al., 2018; Wang et al., 2018). Several separate SNPs in *cpxA* have previously been linked to aminoglycoside (Suzuki et al., 2014) and β-lactam (Dean et al., 2018) resistance following *in vitro* selection. These previously reported SNPs resulted in non-synonymous amino acid changes at the follow positions of Leu-57-Val (Dean et al., 2018), Ala-183-Gly, Ile-95-Phe, Met-22-Arg, and Trp-184-Gly (Suzuki et al., 2014), whereas the non-synonymous SNP we identified within *cpxA* in *E. coli* 10129 was an amino acid change of Pro-Leu at position 177.

Complementation of the SNP within *cpxA* in order to determine functionality in AMC resistance will form the basis of further study.

In a clinical setting, MICs are standardized and are determined in MHB/cation-adjusted MHB according to either CLSI (Clinical and Laboratory Standards Institute [CLSI], 2018) or EUCAST (International Standards Organization [ISO], 2006) guidelines. Various evolutionary studies assess MICs in the same media that is subsequently used for selection of spontaneous AMR mutants, such as M9, ISO, and LB (Farrell et al., 2011; Suzuki et al., 2017; Basra et al., 2018). However, other studies occasionally one medium, such as ISO (Hu et al., 2010) or MHB (Birosova and Mikulasova, 2009), to perform MICs and a different medium to perform subsequent evolutionary experiments, including nutrient broth (Hu et al., 2010) and LB (Birosova and Mikulasova, 2009). We found that media can have a direct effect on the MIC, most notably an obvious drop in MIC when assessed in M9 compared to MHB, LB, and ISO, and there were also more subtle differences between the latter three media. A recent study has found that supplements added to MHB, such as human serum or lung surfactant, directly affected the activity of antibiotics and therefore the determination of the MIC (Kavanagh et al., 2018). While we understand that the use of MHB in a clinical setting is entirely to ensure standardization across different clinical laboratories to be able to monitor AMR, there is currently no comparable standardization to use for evolutionary studies.

We have found that media not only has a direct effect on the selection of spontaneous mutants conferring resistance to AMC in our experiments, but it also affects the competitive fitness of AMC-resistant strains selected for in LB, as has previously been acknowledged (Maharjan and Ferenci, 2017; Lin et al., 2018). Although both LB_2 and LB_5 contain the same mutation in the *ampC* promoter region which conferred resistance to AMC (Jaurin et al., 1982; Caroff et al., 2000), it is likely that either LB_5 has a compensatory mutation that allows it to grow better in ISO and M9 or there is a mutation elsewhere within the genome of the strains which results in a negative (for LB_2) or positive epistatic (for LB_5) interaction(s) only detectable during growth in ISO and M9 (Supplementary Table S1). It is noteworthy that any compensatory mutations or mutations resulting in epistatic interactions identified in the genome would not be able to be linked to the growth phenotype unless comparative fitness assays were carried out in the different media. A similar phenomenon has previously been observed when competitive fitness was assessed in LB, M9, and tryptone soya broth following adaption of *E. coli* K12 MG1655 to LB to select for mutations that compensate for a loss of fitness following a mutation in either *gyrA* or *marR* (Basra et al., 2018). Both this study, ours and other previous studies (Maharjan and Ferenci, 2017; Lin et al., 2018; Yokoyama et al., 2018), highlight that mutations, and the associated fitness effects, can have different consequences in different environments.

To determine if any of the data we derived from growth in laboratory media could have predictive value in a more clinically relevant environment, we assessed growth in urine and urothelial organoids. We found that MICs of the *E. coli* 10129 AMC-resistant isolates, when determined in urine, were not directly comparable to any media type we tested in this study and, more importantly, that the relative fitness of the AMC-resistant isolates was not comparable to relative growth in urothelial organoids. The human bladder urothelial organoid model system used in this study was deliberately chosen as it closely represents the stratified and differentiated bladder urothelium, which is a common, and clinically important, *in vivo* environment for *E. coli* (Horsley et al., 2018). The availability of cellular constituents secreted into the urine from the epithelial cells of the urothelial organoid, as well as nutrients and attachment factors present on the urothelial surface itself, along with its elaboration of a glucosaminoglycan layer, are all likely to have a direct effect on bacterial growth, and therefore affecting the MIC of the bacteria, neither of which would be accurately predicted by growth in laboratory media.

As biofilm-producing bacteria are a major cause of catheter-associated UTIs, of which *E. coli* is among the most common cause (Sabir et al., 2017), we sought to determine whether there is any association between biofilm formation, MICs, and fitness assessed in the different growth media, including urine and urothelial organoids. We found no correlation between fitness, MICs, and biofilm production in our *E. coli* 10129 derivative strains suggesting that this lack of predictive value between MIC and fitness in different media is also evident between MIC, fitness, and biofilm forming ability for this strain.

We acknowledge there are limitations to this study, not least the limited amount of different *E. coli* isolates, bacterial strains, and antimicrobials tested, and the limited number of AMC-resistant derivative isolates tested following selection. While we had a very limited number of fully susceptible clinical isolates from Malawi to choose from Musicha et al. (2017b) we consider the use of multiple evolutionary lineages derived from a single strain sufficient to demonstrate variability and reflects the evolving epidemiological landscape of pathogenic *E. coli* (Manges et al., 2001; Yamaji et al., 2018).

## Conclusion

These results highlight the importance of not only assessing aspects of evolutionary studies in several media types, but also the importance of using *in vitro* model systems, such as urothelial organoids, to ensure the phenomenon that is observed occurs in a range of different settings rather than in a single environment. For example, a diagnostic laboratory using MHB to determine sensitivity of the ISO-derived strains in this study would have deemed them resistant to AMC, whereas the MIC in urine would report that the strains were actually sensitive. If the MHB-derived information were deployed in a clinical setting, this could lead to incorrect treatment of the patient, resorting to a less-optimal alternative or delaying the administration of a more suitable drug. Indeed, in clinical experience, mismatches between predicted and real-world response outcomes of antibiotic treatment have been observed; our results could in part explain these discrepancies.

## Accession Numbers

This assembled genome of the ancestral isolate, *E. coli* 10129, has been deposited at GenBank under the accession SIJF00000000. The version described in this paper is version SIJF01000000. The sequencing reads of the six AMC-resistant isolates derived from the ancestral isolate and submitted under the accession number PRJNA522956.

## Data Availability

The datasets generated for this study can be found in GenBank, SIJF00000000 and PRJNA522956.

## Author Contributions

AH, NJ, NF, JR, and AR contributed to the experimental design and data analysis. AH and AR contributed to the conceptualization and writing the first draft of the manuscript. AH and NJ contributed to carrying out the experiments.

## Conflict of Interest Statement

The authors declare that the research was conducted in the absence of any commercial or financial relationships that could be construed as a potential conflict of interest.
